# Ovarian stimulation medications and patients’ responses as prognostic factors in IUI-treated infertile Saudi patients 

**Published:** 2014-07

**Authors:** Ahmed M. Isa, Basim Abu-Rafea, Sahel Al-Asiri, Johara Al-Motawa, Khalid Almady, Raheek Alwaznah

**Affiliations:** *Assisted Conception Unit, Department of Obstetrics and Gynecology, College of Medicine, King Saud University, Riyadh, Saudi Arabia.*

**Keywords:** *Artificial Insemination*, *Ovulation induction*, *Ovarian Follicles*, *Pregnancy Rate*

## Abstract

**Background:** Intrauterine Insemination (IUI) remains the first thought of infertility treatment.

**Objective:** To compare the stimulation effects and Pregnancy rate (PR) outcomes of two ovulation induction (OI) medications, human-derived menopausal gonadotrophins (hMGH), Merional (MER), and recombinant follicular stimulating hormone (rFSH), Puregon (PUR), in a cohort of Saudi infertile patients, for better predictability of treatment results.

**Materials and Methods: **During a 24-month period, 296 women underwent IUI single treatments. PR’s were correlated with the type of stimulation medication that were prospectively and randomly assigned to each patient, and with the number and size of maturing follicles detected on the hCG injection day.

**Results: **MER and PUR needed comparable number of days (9.26±4.74 and 9.73±6.27 respectively) before follicles were ready for IUI, although the average amount used from MER, 1199.90 IU, was about double that was used from PUR, 621.08 IU. The overall PR in case of PUR however was nearly double that of MER, 13.28% and 7.14% respectively. The best PR, 16.22%, occurred when the follicles matured within 12-13 days. Three follicles of at least 15-mm diameter on the hCG day had better PR’s than one or two, however when the follicles’ diameters were at least 18-mm, PR was significantly higher, (p=0.013).

**Conclusion:** MER and PUR had comparable stimulation effects; however PUR had noticeably higher PR. The best PR occurred when the follicles matured within 12-13 days. PR in case of three maturing follicles on the hCG day was better than only one or two, and significantly better when their diameters were at least 18 mm.

## Introduction

The advantages of Intrauterine insemination (IUI) treatment, with and without mild ovarian stimulation, were early recognized and applied for the treatment of patients with mild male factor, cervical factor, immunological factor, or unexplained infertility reasons (1). In addition, IUI has been shown to be much less expensive and much less invasive procedure in comparison with the in vitro fertilization-embryo transfer (IVF-ET) procedure (2). Pregnancy rates (PR’s) variation was so wide, based on the differences between the studied groups of patients and the variation of the studied parameters (3-8). In general, results from various studies indicated a high degree of consistency, however some variations were evident which could be explained through the population differences and circumstances variability under which each study was conducted (1). 

The objective of this prospective study was to compare the stimulation effects and results including the PR outcomes of two ovulation induction (OI) medications, the human-derived menopausal gonadotrophins (hMGH), Merional (MER), and the recombinant ovulation induction follicular stimulating hormone (rFSH), Puregon (PUR), after single IUI treatments, in a cohort of Saudi infertile patients, with variable causes of infertility, for better predictability of treatment results. We studied the effect of the above ovulation stimulation medications on the patients’ responses in terms of the number of days needed for their follicles to mature, and the number and size of the maturing follicles on the hCG day in relation to the PR’s outcome. We wanted to find out how different or similar these OI medications were.

## Materials and methods

In this prospective randomized study that was carried out for 24-month period; January 2009 till October 2010, in the Assisted Conception Unit of King Khalid University Hospital in Saudi Arabia, after the approval of the Institutional Research Bureau of the College of Medicine, patients complained of infertility had undergone comprehensive examination and etiology evaluation for both partners. The examinations included two semen analyses, including the determination of the total motile sperms count (TMSC) before and after semen sample preparation. As needed, further evaluation for the male partners included serum levels of follicular stimulating hormone (FSH) and testosterone. 

For the female partners, different parameters had to be evaluated, like baseline hormonal levels of FSH, luteinizing hormone (LH), thyroid stimulating hormone (TSH), and prolactin. After prescribing the IUI procedure to those who were eligible, and after they signed the study informed consent forms, the gonadotropins stimulation protocol was assigned and explained to the patients. Randomly, the ovulation stimulation medications PUR (Follitropen beta) of Organon (50 IU/day), or MER of IBSA, (75 IU/day) was assigned to every patient. Daily doses were adjusted based on the patient’s response history. Short stimulation induction protocol was applied for all patients, where stimulation starts on the second day of the patient’s menstrual period. The patients were then monitored by ultrasound imaging for better dosage management and for proper timing of insemination. When the leading follicle (s) (no more than three developing follicles) were at least 15-18 mm in diameter, which is a known indicator in most IUI treatment protocols, the patient were instructed to get the injection of 5,000 IU of human chorionic gonadotropin hormone (hCG) exactly 36 hours before the insemination time. Patients with no stimulated follicles after 17 days and patients with more than three stimulated follicles were excluded from the study.

On the insemination day, the semen specimen was centrifuged through two-layered density gradient (PureSperm 40/80 from Nidacon International- Sweden, catalog #: PSK-020), for 20 minutes, at 18000 rcf. The sperm supernatant was then washed twice with HEPES-buffered media (Quinn’s Advantage Medium, with HEPES, from SAGE Company, catalog #: 1023), supplemented with serum (Quinn’s Advantage Serum Protein Substitute SPS from SAGE Company, catalog #: 3010), where final sperm product of each specimen was constituted within 0.5 ml of the same media where it became ready for IUI. The pre- and post-preparation semen parameters were recorded. Cases with less than ten million of total motile sperms post wash were also excluded from the study. The beta hCG pregnancy test analysis was done two weeks after the insemination.

Statistical analysis

The data was then statistically analyzed using the SPSS software (Statistical Package for the Social Sciences, version 16.0, SPSS Inc, Chicago, Illinois, USA). Chi-Square test was used to compare between pregnant and non-pregnant groups with respect to the studied variables. We assumed there was a significant difference when p<0.05.

## Results

**Overall pregnancy rate (PR) comparison**

Two ovulation induction medications were compared (Table I). The first stimulation medication, MER, was used by 56.76% of the patients and it resulted in a PR of 7.14%. The second medication, PUR, was used by 43.24% of the patients and it resulted in 13.28% PR (p=0.075). MER average units used per patient was 1199.90 (Range: 225-7950), while PUR average units used per patient was 621.08 (Range: 140-2450).

**Day’s stimulation lasted before IUI**

Generally, the days of ovulation induction (OI) for both medications and for all groups tested were very comparable with no significant difference between all groups. For the first group, 31.08% of the patients, OI lasted for 6-7 days and resulted in PR of 8.70% (Table I). The second group, 27.36% of the patients, needed 8-9 days and resulted in PR of 9.88%. 

In 20.95% of the patients, OI lasted for 10-11 days, and the PR was 6.45%. Whereas in 12.50% of the patients the OI lasted for 12-13 days and they had the highest, though not yet significant, PR of 16.22% (p=0.067). The PR dropped back to 8.33% when the OI stayed for 14-19 days, and this happened with 8.11% of the patients. The averages of stimulation days were 9.73 and 9.26 for PUR and MER, respectively.

**Number of 15 mm follicles on the hCG day**

Results of both medications were very close and very comparable; however the combined results of both medications were remarkable. 50.34% of all patients had only one 15 mm follicle on the hCG day and they had a PR of 8.72% (Table I). While, 34.79% had two 15 mm follicles and they resulted in a PR of 8.74%. Last category, which was 14.87% of the patients, had three of the 15 mm follicles, and they ended up with a noticeably higher but not yet significant PR of 13.64% (p=0.591). 

**Number of 18 mm follicles on the hCG day**

Results of both medications were again very close and very comparable; however the combined results of both medications were significant. 30.07% of the patients did not have any 18 mm follicles on the hCG day, yet they resulted in PR of 6.74% (Table I). While 54.73% of them had only one 18 mm follicle and they resulted in PR of 9.87%. The following category comprised 13.16% of the patients, and they had two of the 18 mm follicles on the hCG day, and those ended up with a PR of 7.69%. The last category had three of the 18 mm follicles on the hCG day and those comprised only 2.35% of the patients. This group resulted in exceptionally higher and statistically significant PR of 50% (p=0.013).

**Table I:** Ovarian stimulation medication, days of treatment, and the number of 15 mm, or 18 mm follicles on the hCG day; numbers and percentages of patients and the corresponding pregnancy rates after IUI treatment in each category

**Parameter**					**Number of patients (%)**	**Pregnancies Number (%)**	**p-value**
Stimulation Medication							0.075
				Merional	168 (56.76)	12 (7.14)	
				Puregon	128 (43.24)	17 (13.28)	
Days of Treatment							0.067
		6-7 days			92 (31.08)	8 (8.70)	
		8-9 days			81 (27.36)	8 (9.88)	
		10-11 days			62 (20.95)	4 (6.45)	
		12-13 days			37 (12.5)	6 (16.22)	
		14-19 days			24 (8.11)	2 (8.33)	
No. of 15 mm follicles							0.591
			One follicle		149 (50.34)	13 (8.72)	
			Two follicles		103 (34.79)	9 (8.74)	
			Three follicles		44 (14.87)	6 (13.64)	
No. of 18 mm follicles							0.013
	None				89 (30.07)	6 (6.74)	
	One follicle				162 (54.73)	16 (9.87)	
	Two follicles				39 (13.16)	3 (7.69)	
	Three follicles				6 (2.03)	3 (50.00)	

MER average units used per patient was 1190 (Range was 225-7950), while PUR average units used per patient was 625 (140-2450), Chi-square test.

## Discussion

The highly purified human-derived menopausal gonadotrophins (hMGH), which contains a balanced mix of hFSH and hLH, like MER, were known and used first for the OI in women for infertility treatment. Then later, recombinant forms of both hormones, rFSH, like PUR, and rLH, were used, either separately or in mixtures. The comparative studies between those natural and recombinant OI medications will continue to occur for the best of the patients. Here we were compared between MER and PUR after they used for IUI treatment for a group of patients.

More than half of the patients in our study (56.76%) used MER with a PR of 7.14% (Table I), while the rest of them (43.24%) used PUR and had a considerably higher but not yet significant PR, 13.28% (p=0.075). This was in spite of the fact that the average MER units used per patient (1190.90 IU, 225-7950 range) was almost double the average PUR units used per patient (625 IU, range 140-2450). The relatively high average units used from both medications because of the relatively high percentage of poor responders our clinic usually treats. These results are in line with Petanoveski *et al* and Henk *et al* who stated that fewer dosages of rFSH were needed to induce more effect than from HP-hMG, with probably more eggs and better embryos, though there was no significant difference in the PR (8, 9). These results were slightly different from those of Kocak *et al* whose data suggested that HP-hMG and rFSH might be equally suitable for mild ovarian stimulation, followed by IUI (10). Our findings were completely in line with the study of Freour *et al* who had a clinical pregnancy rate of rFSH that was more than double that of the HP-hMG (7). These findings though, were not in line with the findings of Sagnella *et al* who stated that HP-hMG was not at all inferior, compared with rFSH regarding clinical PR (11). 

The range of days OI lasted before the developing follicles were ready for insemination was broad; 6-15 days, however, both medications had very comparable results in this aspect. We dissected that range into small-ranged groups considering the number of patients in each group. The first three groups (where the days of stimulation were 6-7, 8-9, and 10-11 days, respectively), comprised together 79.19% of the total number of the patients, and they had a comparable success rate of 8.70%, 9.88%, and 6.45% respectively (Table I). Then the fourth group of patients received their stimulation medication for 12-13 days, about the same scenario in natural pregnancies in women with regular 28-day menstruation cycles; the PR climbed up to 16.22% (p=0.067). 

For this group which comprised 12.5% of the total number of patients, probably the reason for this noticeably higher PR could be that the uteruses probably had better chances to get ready for embryo implantation, along with a suitable luteal phase hormonal levels in the blood circulation. These findings are in exact agreement with Khalil *et al* who stated that the best window for IUI insemination for the best results they achieved was between the thirteenth and the sixteenth (12). The last group took their medication for 14-19 days and had a PR of 8.33%, which was comparable to the first three groups. This might be because the optimal conditions for embryo implantation were just starting to fade out. This sequence of the above results is in line with pregnancies of the natural and regular 28-day cycles and the expected window of readiness of the uterus lining to receive and accommodate the new fetus. The presence of one or two of the 15 mm follicles on the morning of the hCG injection day, i.e. two days before the IUI, did not make any difference. 

A percentage of 50.34% of the patients had one 15 mm follicle, while 34.79% had two of them; yet the PR for both was almost the same, 8.72% and 8.74%, respectively (Table I). While, with 14.87% of the patients who had three of the 15 mm follicles on the hCG injection day, they had a conspicuous, but not yet significant increase in their PR, 13.64%. It is worth mentioning that none of these cases resulted in triplet fetuses; and less that 5% had twins. That gives an advantage to patients who grow 2-3 follicles and not one. This result is exactly in line with what Zadehmodarres *et al* found (13). The same result also was stated by the survey done by Rawal *et al* on 150 reproductive clinics in UK exploring their attitudes towards the factors that affect the IUI results (14). The highest score was to the number of developing follicles on the hCG day. 

In addition, it was reported that a significant increase in PR per cycle with patients who developed three follicles at the hCG injection time (15). Aiming at three growing follicles before IUI, though has the advantage of a higher conception potential, but might carry the risk of multiple pregnancies. In our unit, more than two developing follicles of at least 14 mm on the hCG day is very discouraged for IUI, and is restricted to cases with specific circumstances; for example, poor responders who failed the stimulation before. This is in agreement with Khalil *et al* who found that unacceptable multiple pregnancy results were expected with four or more growing follicles (12). More data need still to be collected in this regard. This data is also in line with Merviel *et al* who found that two, and not one, 16 mm follicles on the hCG day were better to aim at for IUI cases for achieving significantly better results (16). 

When we studied the effect of having 18 mm follicles on the hCG injection day, the first group constituted 30.07% of the patients who had no follicles of 18 mm on the hCG day (but had at least one follicle of 15 mm diameter), with PR of 6.74% (Table I). While in the second group of 54.73% of the patients, each patient had only one of the 18 mm follicles on the hCG day, and the PR was 9.87%. With the third group (13.16% of the patients), who had two of the 18 mm follicles on the hCG day, the PR of 7.69%, a comparable result with the previous two groups was recorded. 

On the other hand, in the last group (6 patients or 2.03% of the total), there were three follicles of 18 mm follicles on the hCG day and the PR significantly was increased 50% (p=0.013). Keeping in mind that when those three follicles were only 15 mm on the hCG day, the PR was only 13.64%, which means that both number and maturation level of the follicles on the hCG day do matter. Apparently, three maturing follicles on the hCG day is the magic number for IUI treatment, yet they have to be around the 18 mm diameter. This is in line with the survey results of Rawal *et al* who stated that the second factor that people voted for, when it comes to factors that affect the pregnancy results of IUI, was the size that the growing follicles reach on the hCG injection day (14). 

We add that the follicles number is just as significant, while three ready follicles must be restricted to specific cases, poor responders, previous muli-failure, and patients who are older than forty, for avoiding the multiple pregnancy risk. It is worthy to mention that as far as the sizes that the developing follicles gained by the hCG day, both medications used had very comparable results among each group with no apparent difference or preference to any of the two medications.

## Conclusion

The study showed that the ovarian stimulation results of the highly purified human-derived menopausal gonadotropins (hMH), MER, and the recombinant ovulation-induction hormone (rFSH), PUR, were very comparable. However the PR of PUR was close to double that of MER. Days of ovulation stimulation that were analogous to natural regular cycles, i.e. 12-13 days, resulted the best PR. The study showed also that both the follicles’ numbers and sizes were as significant factors. Three follicles of 18 mm size on the hCG day gave the best significant PR result, but this should be restricted to some special prognostic characteristics as mentioned earlier.
